# Surface-Functionalized Metal–Organic Frameworks for Binding Coronavirus Proteins

**DOI:** 10.1021/acsami.2c21187

**Published:** 2023-02-14

**Authors:** Aamod V. Desai, Simon M. Vornholt, Louise L. Major, Romy Ettlinger, Christian Jansen, Daniel N. Rainer, Richard de Rome, Venus So, Paul S. Wheatley, Ailsa K. Edward, Caroline G. Elliott, Atin Pramanik, Avishek Karmakar, A. Robert Armstrong, Christoph Janiak, Terry K. Smith, Russell E. Morris

**Affiliations:** † EastChem School of Chemistry, University of St Andrews, North Haugh, St Andrews KY16 9ST, U.K.; ‡ School of Biology, University of St Andrews, Biomedical Sciences Research Complex North Haugh, St Andrews KY16 9ST, U.K.; § Institut für Anorganische Chemie und Strukturchemie, Heinrich-Heine-Universität Düsseldorf, 40204 Düsseldorf, Germany; ∥ Department of Chemistry, University of Pennsylvania, Philadelphia, Pennsylvania 19104-6323, United States of America

**Keywords:** SARS-CoV-2, antiviral drugs, metal−organic framework, protein binding, water adsorption

## Abstract

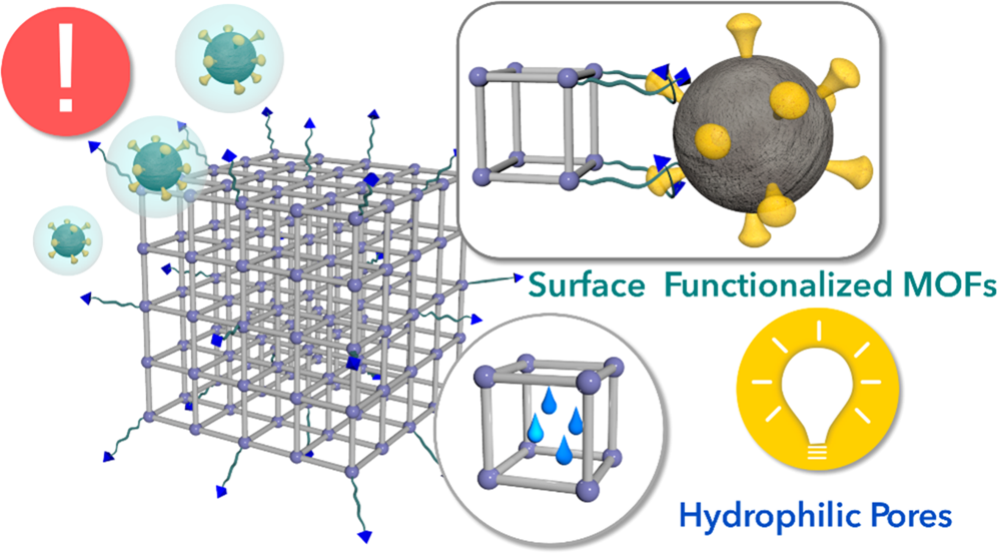

Since the outbreak of SARS-CoV-2, a multitude of strategies have been explored for the means of protection and shielding against virus particles: filtration equipment (PPE) has been widely used in daily life. In this work, we explore another approach in the form of deactivating coronavirus particles through selective binding onto the surface of metal–organic frameworks (MOFs) to further the fight against the transmission of respiratory viruses. MOFs are attractive materials in this regard, as their rich pore and surface chemistry can easily be modified on demand. The surfaces of three MOFs, UiO-66(Zr), UiO-66-NH_2_(Zr), and UiO-66-NO_2_(Zr), have been functionalized with repurposed antiviral agents, namely, folic acid, nystatin, and tenofovir, to enable specific interactions with the external spike protein of the SARS virus. Protein binding studies revealed that this surface modification significantly improved the binding affinity toward glycosylated and non-glycosylated proteins for all three MOFs. Additionally, the pores for the surface-functionalized MOFs can adsorb water, making them suitable for locally dehydrating microbial aerosols. Our findings highlight the immense potential of MOFs in deactivating respiratory coronaviruses to be better equipped to fight future pandemics.

## 1 Introduction

The primary mode in airborne transmission of any respiratory virus, such as SARS-CoV-2, is droplets or aerosols produced by an infected person from actions, such as breathing, speaking, coughing, or sneezing.^1^ The scale of the COVID-19 pandemic^2^ has led to extensive research into understanding the generation, transmission, deposition, and survival of virus-laden aerosols and the factors affecting them. Social distancing and the use of filtration devices, such as PPEs and air purifiers, have been established as a vital line of defense.^3−5^ Although such devices have continuously been researched, the lessons from the ongoing pandemic necessitate their further development to ensure better preparedness for any future airborne diseases.^6^

Filtration devices, such as face masks, are designed to sieve virus aerosols or droplets; however, their performance can be significantly improved by adding functional layers to selected appropriate fabrics.^7^ In addition to size-selective physical filtration, the use of an electrostatic or polar surface helps to attract and trap viruses to prevent further transmission,^8−11^ but at the same time, the material should not compromise breathability.^7^ Metal–organic frameworks (MOFs) have gained attention in this area, as they are well-known adsorbents. MOFs, which consist of inorganic and organic building units, feature an immense design variability and consequently offer myriad applications, including the possibility of integrating them into composites or textiles toward real-world utility.^12−14^ In addition, MOFs can be functionalized externally,^15−17^ to manipulate their surface wettability and roughness.^18,19^ These features complement the desirable properties for a hydrophilic surface that exhibits higher uptake of water,^20^ improved filtration efficiency,^21^ and the ability to significantly reduce the drying time of virus-bearing aerosols to inactive the pathogen.^22^ Owing to their porosity,^23^ we propose an approach to combat airborne viruses using MOFs with specific antiviral functionalization. The appropriate surface modification would ensure high binding affinity with the external layer of viruses, while water adsorption^24^ by the MOF particles conceivably causes local dehydration of the aerosols.^25^ The proposed approach truly taps the full potential of MOFs—tunable surface and pore chemistry—and further drives the growing research on antimicrobial filters based on MOFs.^26−30^

The pathogens in the coronavirus family exhibit a similar structure with an extruded spike protein (S-protein) that is responsible for binding to human cell receptor—angiotensin-converting enzyme 2 (ACE2).^31−33^ Both vaccine and therapeutic approaches for tackling these viruses primarily target the S-protein.^34^ The S-protein of coronaviruses is a glycoprotein that comprises amino acid residues in its receptor binding domain (RBD). Extensive in silico drug repurposing studies have been reported, in addition to developing new drugs, highlighting several candidates with effectiveness against coronaviruses.^35,36^ It is worth noting that some of these drugs also showed binding ability to the S-protein of other coronaviruses or even the variants of SARS-CoV-2.^37^ Nevertheless, there are still a few bottlenecks for the practical implementation of these drugs, and some of them have not been effective in clinical trials.^38^ However, the terminating interface of MOF particles provides a well-defined chemistry that enables binding with carboxylate or phosphonate groups in certain drugs from repurposing studies. This has been shown in different MOFs where a successful surface functionalization of MOFs occurred through coordination with the metal nodes on the surface of MOFs.^16^

**Scheme 1:**
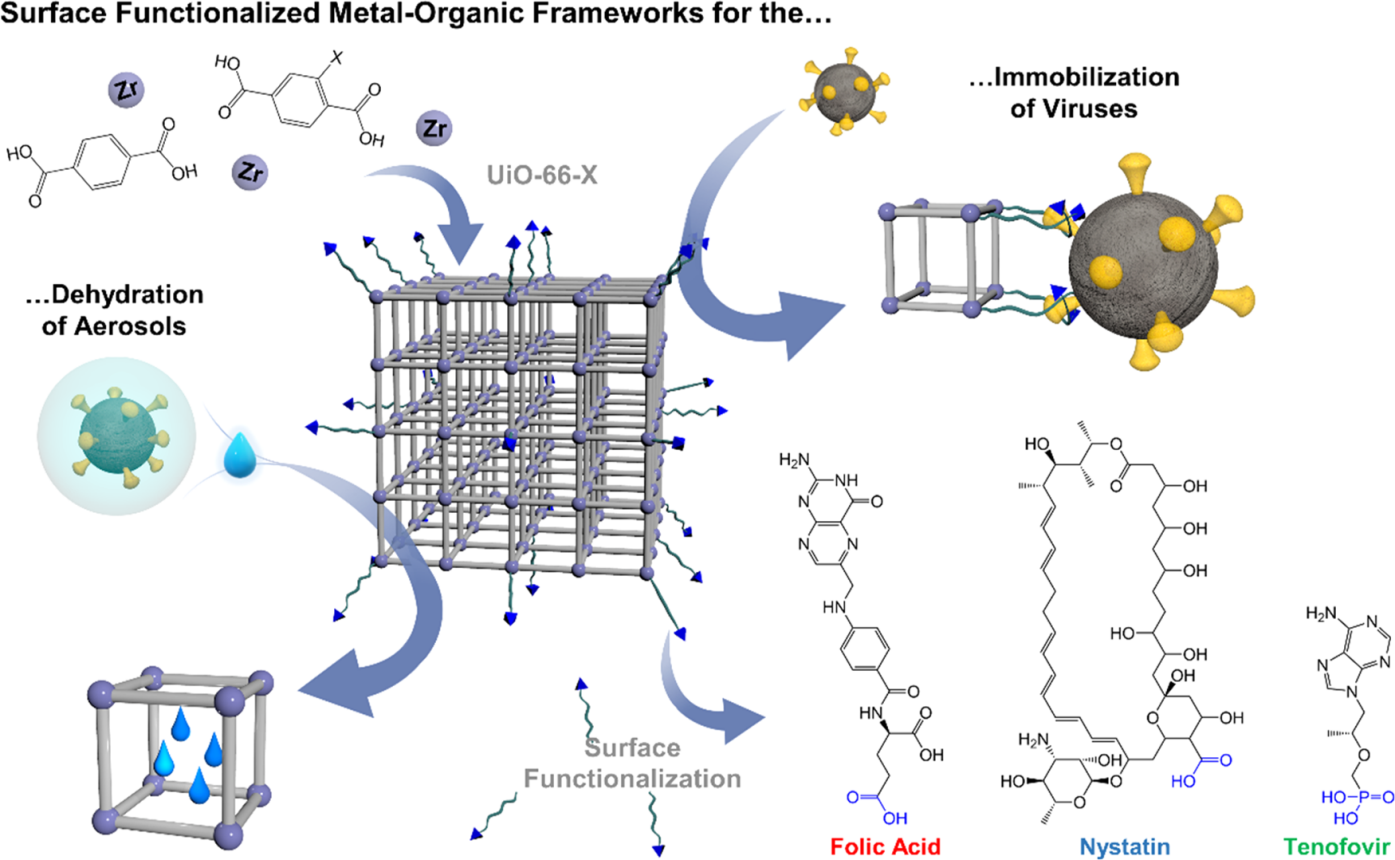
Schematic Representation of the Utilization of Different MOFs, Namely, UiO-66-X (X = −H, NH_2_, NO_2_), with Hydrophilic Pores for Dehydrating Aerosols/Droplets and Different Surface Functionalization for Immobilizing Viruses, i.e., Folic Acid, Nystatin, and Tenofovir with the Expected Binding Groups to the MOF Surface Highlighted in Blue

Combining these hypotheses, we report the surface functionalization of three Zr(IV)-based UiO-66 MOFs (UiO-66, UiO-66-NH_2_, and UiO-66-NO_2_) using three repurposed agents against COVID-19, i.e., folic acid (FA),^39^ nystatin (Nys),^40^ and tenofovir (Teno),^41^ as a model system to lay the groundwork for the continued development of novel agents to deactivate coronavirus particles and highlight the correlation of surface chemistry to binding of proteins with the ultimate aim of developing MOF-based antiviral filters/coatings (Scheme 1). The pores of the MOFs can adsorb water even after functionalization, and the functionalized compounds exhibit significantly higher affinity over pristine MOFs toward all of the tested proteins—glycosylated bovine serum albumin (BSA), non-glycosylated Annexin-g03104, and the S-protein of the actual SARS virus.

## 2 Experimental Section

### 2.1 Materials

All chemicals—zirconium tetrachloride (Sigma-Aldrich), terephthalic acid (Sigma-Aldrich), 2-amino terephthalic acid (Sigma-Aldrich), 2-nitroterephthalic acid (Fluorochem), hydrochloric acid (Fisher Scientific), *N*,*N*′-dimethyl formamide (Acros Organics), ethanol (VWR), methanol (Fisher Scientific), hexane (Sigma-Aldrich), folic acid (Sigma-Aldrich), nystatin (Acros Organics), and tenofovir (TCI Chemicals) were obtained from commercial sources and used without purification. The proteins—BSA (Fisher Scientific, standard grade powder heat shock treated) and recombinant SARS spike protein (ab49046, Abcam)—were also purchased from commercial suppliers, while Annexin-g03104 was expressed and purified in-house. The Annexin-g03104 protein was recombinantly expressed with an N-terminal His-tag, which was removed with Tev protease during purification.^42^

### 2.2 Synthesis of MOFs

All UiO-66-series MOFs (UiO-66, UiO-66-NH_2_, UiO-66-NO_2_) were synthesized using a reported protocol.^43^ The general procedure involved adding calculated amounts of ZrCl_4_ (0.5 g, 2.145 mmol), DMF (20 mL), and concentrated HCl (4 mL) to a glass jar with PTFE-coated screw caps. The mixture was sonicated for 20 min. The respective ligand (3.0 mmol) and DMF (40 mL) were added, and the mixture was further sonicated for 20 min. Following that, the jar was left in an oven heating at 80 °C for 24 h. Upon cooling, the sample was filtered and washed with DMF (3 × 30 mL), EtOH (30 mL), and hexane (15 mL). The recovered solid was dried in an oven at 80 °C overnight.

### 2.3 Postsynthesis Functionalization

The procedure was adapted from previous reports.^44,45^ The functional molecule [folic acid (FA), nystatin (Nys), or tenofovir (Teno)] was dissolved or dispersed in respective solvents and added dropwise under stirring to a suspension of the parent MOF (0.125 g) in MeOH. The mixture was left to stir for 24 h at room temperature. Subsequently, the solid was recovered by centrifugation (6000 rpm, 20 min) and washed with respective solvents. The product was dried in an oven at 80 °C overnight.

The masses for different functionalization reactions are shown in Table 1. The solvents used for the functionalization reactions are shown in Table 2.

**Table 1:** Masses for Different Functionalization Reactions

MOF (0.125 g)	mass of FA (g)	mass of Nys (g)	mass of Teno (g)
UiO-66	0.0820	0.137	0.0531
UiO-66-NH_2_	0.0806	0.135	0.0524
UiO-66-NO_2_	0.0713	0.119	0.0464

**Table 2:** Solvents Used for the Functionalization Reactions

step	MOF-FA	MOF-Nys	MOF-Teno
synthesis	MOF (MeOH, 15 mL); FA (DMF, 20 mL)	MOF (MeOH, 15 mL); Nys (DMF, 5 mL + MeOH, 10 mL)	MOF (MeOH, 5 mL); Teno (water, 15 mL + MeOH, 5 mL)
work up	DMF (2 × 20 mL), MeOH (1 × 20 mL), hexane (1 × 20 mL)	DMF (2 × 20 mL), MeOH (1 × 20 mL), hexane (1 × 20 mL)	water (2 × 20 mL), MeOH (2 × 20 mL)

### 2.4 Structural Characterization

Powder X-ray diffraction (PXRD) patterns were recorded on an STOE STADI/P diffractometer using Cu K_α1_ radiation at room temperature from 3 to 50° (2θ). Thermogravimetric analysis (TGA) and differential thermal analysis (DTA) were recorded in air on a Stanton Redcroft STA-780 from room temperature to 700 °C, with a heating rate of 5 °C/min. FTIR spectroscopy was carried out using a Shimadzu IR Affinity-1 FTIR spectrophotometer in transmittance mode from 400 to 4000 cm^–1^. Solid-state UV–vis absorption was performed on a JASCO V-650 spectrophotometer at room temperature on solid samples using the diffuse reflection mode. SEM micrographs were collected using a Scios DualBeam at a working distance of 7 mm and low operating voltages (2–5 kV) to ensure sensitive mapping of the surface. The powdered samples were placed on carbon or copper tape and gold coated using a Quorum Q150R ES sputter coater (10 mA, 30 s) prior to recording. TEM micrographs were obtained using an FEI Titan Themis operated at 200 kV on samples prepared by deposition of one drop of the nanoparticle suspension on holey carbon films supported on a 300 mesh Cu grid (Agar Scientific). Raman spectra were recorded on a Renishaw In-Via Qontor Raman microscope using a laser excitation of either 532 or 785 nm. The ζ potential was measured in water and at room temperature using a Malvern Zetasizer μV instrument (Malvern Panalytical, U.K.).

### 2.5 Gas Adsorption

BET specific surface area determination from N_2_ isotherms was carried out according to the Rouquerol theory^46^ using the Microactive Software Kit v4.03.04. Data were recorded on a Micromeritics ASAP 2020 Accelerated Surface Area and Porosity System or on a Micromeritics Tristar ii Surface Area and Porosity Instrument. Samples (∼100 mg) were added to a frit tube and activated in vacuo (120 or 100 °C, ∼3 × 10^–5^ mbar, 16 h) prior to the measurement.

### 2.6 Water Adsorption

Water vapor sorption isotherms were measured volumetrically on a Quantachrome (QUANTACHROME, Odelzhausen, Germany) *VStar4* vapor sorption analyzer with four parallel stations at 293 K. Before each sorption measurement, the samples were activated under vacuum (1 × 10^–3^ mbar) at 383 K for 3 h or until a pressure of 5 × 10^–2^ mbar was achieved, using a *FloVac* (QUANTACHROME, Odelzhausen, Germany) degasser.

### 2.7 Protein Binding Studies

Three proteins were used for protein binding experiments. MOF powders (∼3 mg) were prewashed with autoclaved Milli-Q water. To the recovered solid, 300 μL of water was added and vortexed briefly. The dispersion was put on a rotating wheel for 1 h at room temperature and subsequently centrifuged at 16 k rpm for 5 min. The supernatant was removed and screened for any functional group leaching. Leaching was monitored by measuring supernatant absorption at 280 nm and by testing 50 μL of the supernatant using a MiniBradford assay.

Separately, a 10 mg/mL stock solution of BSA (standard BSA) was prepared in water, its concentration was confirmed using the standard absorbance of A260 = 0.67 for 0.1 mg/mL BSA, and this BSA stock was diluted to either 20 or 200 μg/mL with water. For Annexin-g03104 and SARS S-protein, 200 μg/mL solutions were prepared with water. A 300 μL aliquot of protein solution was added to the MOFs and incubated overnight at room temperature on the rotating table. Thereafter, the samples were centrifuged for 5 min at 16 k rpm. A sample volume of 50 μL of the supernatant was tested using Bio-Rad MiniBradford in triplicate and compared to the BSA standard curve comprising 0, 0.39, 0.78, 1.5625, 3.125, 6.25, 12.5, 25 μg/mL BSA. For 200 μg/mL protein experiments, the supernatant was tested undiluted and diluted to ensure the protein concentration was within the range of the BSA standard curve. The protein concentration in solution was subtracted from the starting concentration to determine the amount bound to the MOFs. Triplicate MOF samples were tested for each binding condition.

## 3 Results and Discussion

**Figure 1:**
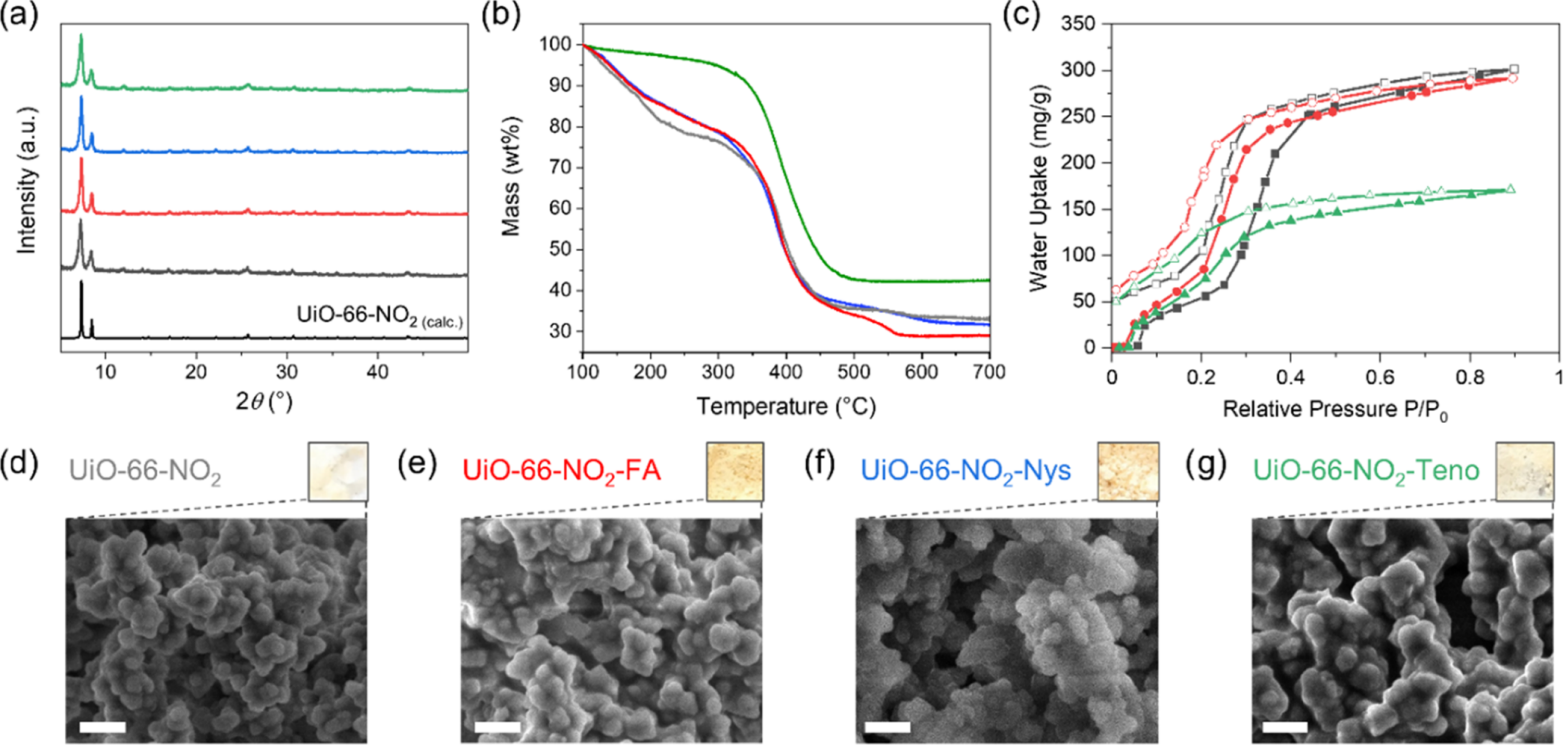
Characterization for surface-modified UiO-66-NO_2_—(a) PXRD patterns, (b) TGA profiles (for a better comparison of their decomposition profiles, the release of different amounts of water due to their respective water adsorption behaviors below 100 °C was omitted), and (c) water adsorption isotherms recorded at 298 K. Photographs and SEM micrographs of (d) UiO-66-NO_2_, (e) UiO-66-NO_2_-FA, (f) UiO-66-NO_2_-Nys, and (g) UiO-66-NO_2_-Teno; scale bar = 500 nm (color code for plots: UiO-66-NO_2_—gray, UiO-66-NO_2_-FA—red, UiO-66-NO_2_-Nys—blue, UiO-66-NO_2_-Teno—green).

MOFs of the UiO-66 family were chosen, as they have been well established for both internal pore and surface functionalization and were synthesized using known reports. The pristine materials were characterized using powder X-ray diffraction (PXRD), Fourier transform infrared spectroscopy (FTIR), and thermogravimetric analysis (TGA) (Figures 1 and S1–S6 and S14). Owing to the presence of terminal carboxylate (FA, Nys) or phosphonate (Teno) groups, these three molecules, i.e., FA, Nys, and Teno, were chosen for surface modification. The coordinative functionalization with FA has been previously established in Zr(IV)-MOFs,^44,45^ and therefore, a similar protocol was employed for the surface modification with all three tag molecules. PXRD patterns (Figures 1a and S1–S3) and FTIR spectra (Figures S4–S6) revealed the complete retention of the MOF structure in all of the cases. Although weak, broad signals in the FTIR spectra for the tag molecules were present in surface-functionalized MOFs (Figures S4–S6), it was not possible to fully validate the surface functionalization. Solid-state UV–vis spectra, however, exhibited discernible broad signals for FA- and Nys-modified MOFs (Figures S7a,b, S9, and S10), consistent with that expected for the individual molecules, even in the case of UiO-66-NH_2_, which has a similar absorption in the UV–vis profile (Figure S9). In the case of Teno, although a weak shoulder was noticeable in the trace (∼390 nm) for both Teno and UiO-66-Teno (Figure S7c), clearer evidence for the presence of the substitution was obtained from its Raman profile with a discernible peak at 724 cm^–1^ and a weak, broad feature between 1310 and 1370 cm^–1^ (Figure S8). Compounds UiO-66-NH_2_-Teno and UiO-66-NO_2_-Teno also exhibited similarly distinguishable signals in the Raman spectra (Figures S11 and S12). Depending on the tag molecule attached, a color change of the MOF powder could also be observed (Figure 1d–g): pristine white UiO-66-NO_2_ became yellowish in the case of orange-yellow FA, pale yellow for smooth yellow Nys, and remained off-white for white Teno.

In addition, scanning electron microscopy (SEM) micrographs were recorded for all pristine MOFs and their functionalized counterparts (Figures 1d–g and S13). The size of the primary MOF particles is approximately 150 nm, and they aggregate to bigger agglomerates ∼5 to 10 μm in size. The overall morphology is retained throughout the functionalization of the MOF (Figure 1d–g). TGA was carried out to estimate the amount of surface functionalization for each MOF (Figures 1b and S14). For a better comparison of their decomposition profiles, the release of different amounts of water due to their respective water adsorption behavior below 100 °C is omitted in this section and discussed in detail later. The TGA profiles of all functionalized MOF compounds are similar to those of their parent MOF. Depending upon the functionalization conditions, i.e., in DMF/methanol for FA and Nys or methanol/water for Teno, a mass loss corresponding to solvent inside the pores of the MOFs can be observed below 300 °C (Figure S14). In all cases (except UiO-66-NH_2_-Nys), the functionalized MOF compounds show an increased weight loss between 300 and 700 °C (Table S1) compared to their parent MOF compound. This can be attributed to the additional decomposition of the respective functionalization and enabled the approximate determination of its amount. The decomposition corresponding to the presence of additional functional groups was also seen in the DTA (differential thermal analysis) trace (Figure S15), where either an extra peak or broadening of the decomposition peak was observed for functionalized compounds. The TGA results indicate an indirect proportionality between the size of the functionalizing moiety and its grafted amount on the MOF surface: with 13.5, 12.0, and 8.3 wt % on UiO-66, UiO-66-NH_2_, and UiO-66-NO_2_, respectively; the amount of Teno, which has the smallest size, was the highest in all cases. With 12.4 and 10.0 wt % FA, which is between Teno and Nys in size, showed the second highest amount for UiO-66 and UiO-66-NH_2_, respectively, while only 2.7 wt % were detected for its functionalization of UiO-66-NO_2_. Nys, which is the biggest functionalizing moiety, showed the lowest amount for UiO-66, no observable amount for UiO-66-NH_2_, and with 6.0 wt %, a higher amount than FA for UiO-66-NO_2_.

Low-temperature (77 K) N_2_ adsorption measurements exhibited similar Type-I profiles for UiO-66 and its derivatives, with lower uptakes and surface areas for the surface-modified compounds (Figure S16 and Table S2). To further examine the accessibility of the pores after the surface modification and to verify one objective of this work, water adsorption isotherms were recorded at 298 K for all of the FA- and Teno-functionalized compounds studied in this work (Figures 1c and S17). Nys-functionalized MOFs were not chosen for these measurements, as they exhibited lower binding to the test proteins, as discussed later. The general trend of the water uptake of the functionalized UiOs is on the order of UiO-66-X > UiO-66-X-FA > UiO-66-X-Teno (X = −H, NH_2_, NO_2_; Figures 1c and S17 and Table S3) and follows roughly the trend of porosity in the sample (Table S2) modulated through the hydrophilicity/hydrophobicity. The modulation, for example, led to a similar total water uptake for UiO-66 and UiO-66-NH_2_ (Figure S17d) and UiO-66-NO_2_ and UiO-66-NO_2_-FA (Figure S17c) through the introduction of more hydrophilic −NH_2_^47,48^ and FA. FA and Teno surface functionalization retained the S-shaped water uptake curves of UiO MOFs. At the same time, a comparison between the experimental water uptake at *pp*_0_^–1^ = 0.9 and the estimate for the composite, based on the wt% of UiO-66-X, reveals considerable pore-blocking effects for the Teno-functionalized compounds UiO-66-Teno, UiO-66-NH_2_-Teno, and UiO-66-NO_2_-Teno (Table S3). However, in the low-pressure region (*pp*_0_^–1^ < 0.2), the hydrophilicity of Teno overcompensates the pore blocking and for UiO-66-NO_2_ leads to an even higher water uptake than the pristine MOF. Also, the increased hydrophilicity through FA functionalization strongly increases the water uptake below *pp*_0_^–1^ = 0.2 for the less-hydrophilic UiO-66-NO_2_ (Figure 1c and Table S3).

However, UiO-66 functionalization with NH_2_ and NO_2_ as well as with FA and Teno induced shifts of the uptake to lower/higher relative pressure when compared to (non-functionalized) UiO-66 or the parent UiO-66-X, respectively. A shift to ‘earlier’ uptake, i.e. to lower relative pressure, means that the functionalization has increased the hydrophilicity, and a shift to ‘later’ uptake, i.e. to higher relative pressure, means that the system has become more hydrophobic compared to a reference. For UiO-66, the introduction of the amino groups increases the hydrophilicity, and NO_2_ decreases the hydrophilicity (Figure S17d), as seen before.^47,48^ The −NO_2_ group even induces a gate-opening effect, that is, requires a minimum pressure of *pp*_0_^–1^ ≈ 0.05–0.07. Further, FA shifted the steep increase of water uptake significantly to higher (for UiO-66 and UiO-66-NH_2_) or lower relative pressure (for UiO-66-NO_2_; Figure S17a–c). Teno, on the other hand, followed the uptake curve of the parent MOF in the low-pressure region with only a slight shift to higher (for UiO-66 and UiO-66-NH_2_) and lower uptake pressure (for UiO-66-NO_2_; Figure S17a–c). Thus, we can assume that the hydrophilicity of FA and Teno lie between those of more hydrophilic UiO-66 and UiO-66-NH_2_ on one side and less-hydrophilic UiO-66-NO_2_ on the other side.

**Figure 2:**
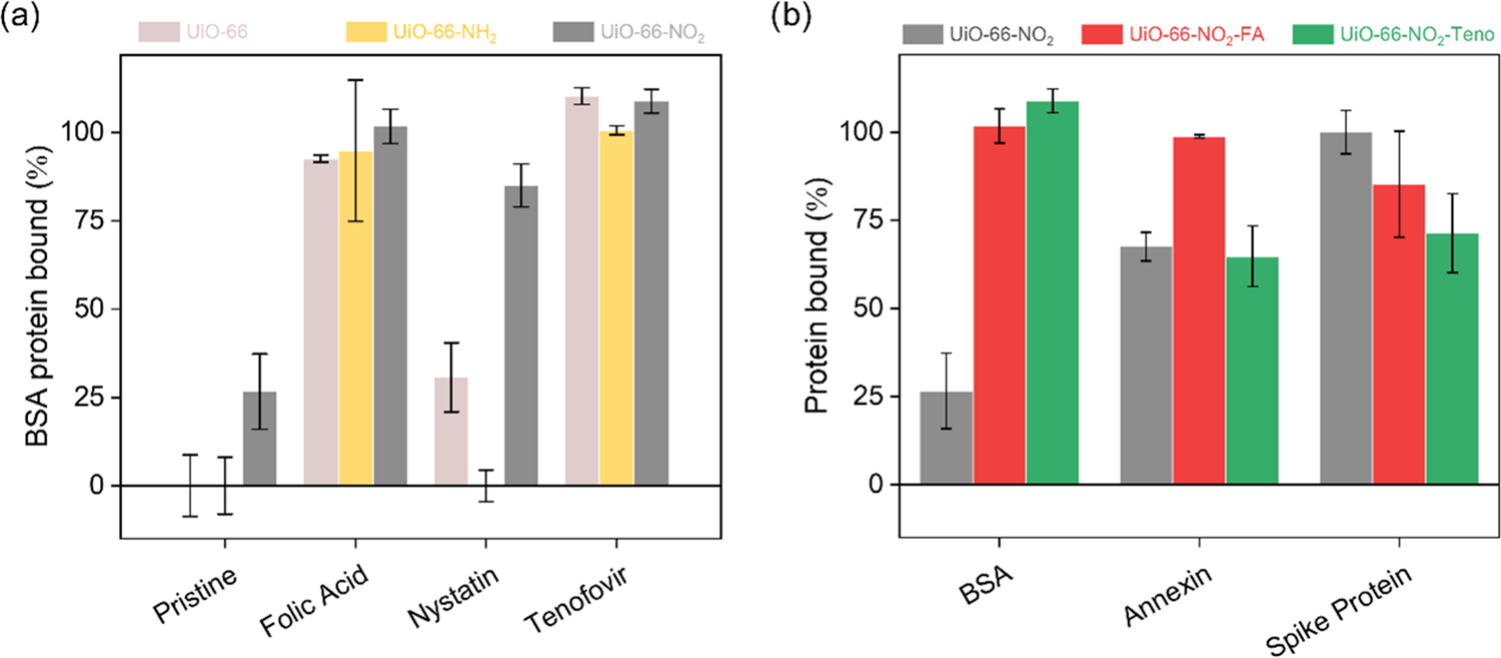
(a) Amount of BSA protein binding (%) when treated with 6 μg of BSA in aqueous solution. (b) Comparison of protein binding (%) of UiO-66-NO_2_ and its derivatives (UiO-66-NO_2_-FA and UiO-66-NO_2_-Teno) upon treatment with 60 μg of BSA, Annexin-g03104, and recombinant S-protein of SARS in water (plots are an average value of triplicate measurements, error bars represent standard deviation).

**Figure 3:**
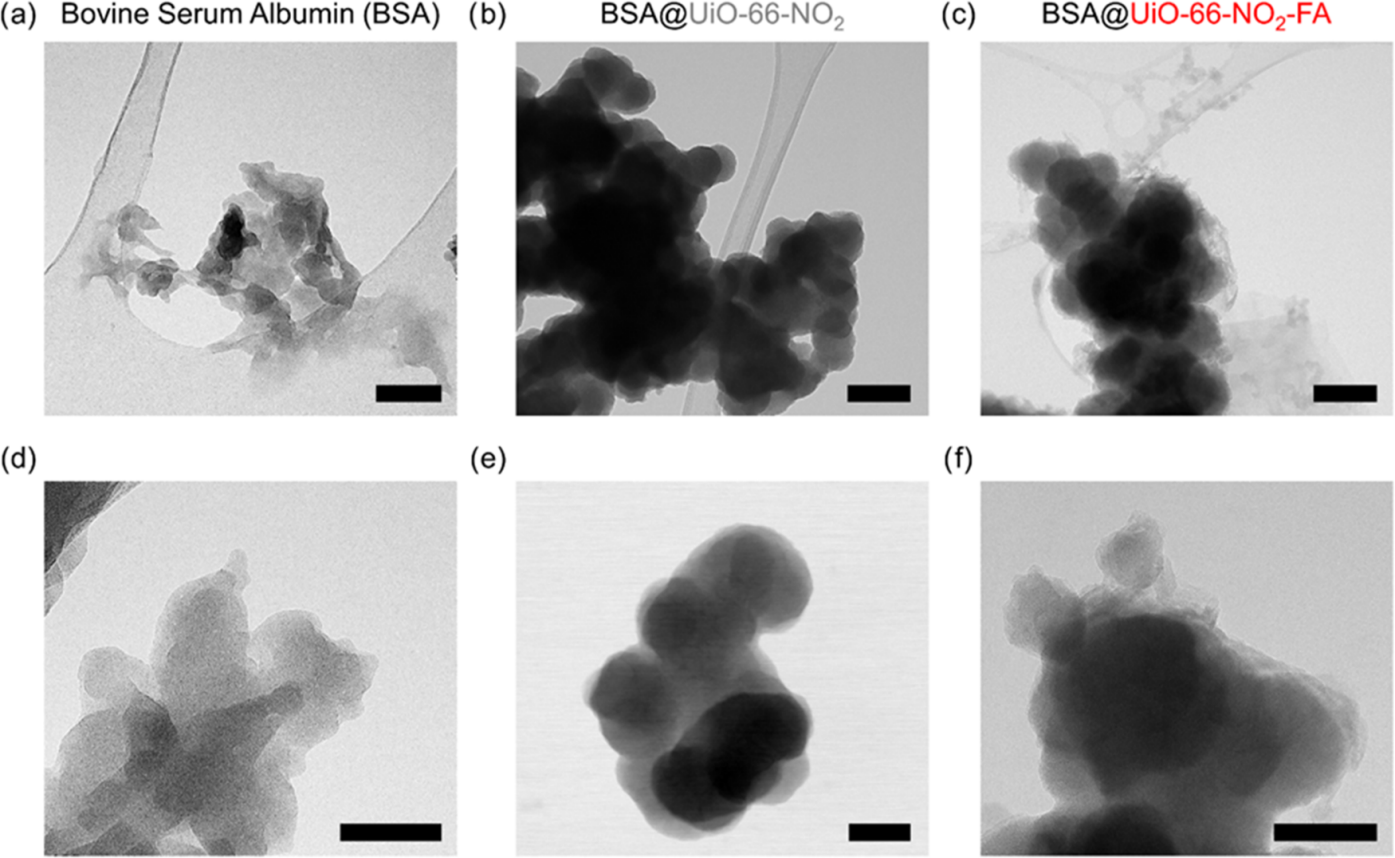
TEM micrographs of bovine serum albumin (BSA; (a, d)), as well as pristine UiO-66-NO_2_ (b, e) and UiO-66-NO_2_-FA (c, f) after the BSA protein binding experiment. Scale bar: (a–c) 200 nm and (d–f) 100 nm.

Subsequently, the ability of the surface-modified MOFs to bind proteins was tested with three proteins: glycosylated bovine serum albumin (BSA), non-glycosylated Annexin-g03104, and the recombinant S-protein of the SARS virus. Initial binding was investigated with BSA as a readily available glycosylated protein. To confirm that the MOF protein binding was not specific to BSA, MOFs were also tested with heterogeneously expressed Annexin-g03104 as a soluble protein tolerant of low salt conditions^42^ and a portion of the SARS virus S-protein. Initially, BSA protein solutions were prepared in water (20 μg/mL), and all of the compounds were treated with the solution containing 6 μg of BSA. Among pristine MOFs, UiO-66 and UiO-66-NH_2_ exhibited negligible BSA binding, while for UiO-66-NO_2_, ∼25% of the protein was bound, suggesting the participation of polar secondary groups (−NO_2_) in the linker for protein binding. MOFs with FA and Teno functionalization had significantly higher affinity over the base MOFs in all of the cases, while Nys-functionalized MOFs showed marginal improvements (Figure 2a). Nys-functionalized MOFs had the least binding among all functionalized solids, corroborating the previous observation of low loadings of Nys functionality, especially in the case of UiO-66-NH_2_. To further understand the influence of the −NO_2_ group, the same protein binding experiment was carried out with a higher concentration of BSA (60 μg at 200 μg/mL; Figure 2b). It was found that the functionalized MOFs—UiO-66-NO_2_-FA and UiO-66-NO_2_-Teno—had a substantially higher affinity (∼100%) over the parent MOF—UiO-66-NO_2_ (∼25%). These findings could be visualized by analyzing the respective samples after BSA protein binding studies with transmission electron microscopy (TEM; Figure 3). In the case of pristine UiO-66-NO_2_, no BSA was present on the surface of the nanoparticles (Figure 3d,e), while its FA derivative clearly evidenced protein binding (Figure 3c,f). Encouraged by these findings, the study was extended to non-glycosylated Annexin-g03104 and the recombinant S-protein of the actual SARS virus using the best-performing MOFs—UiO-66-NO_2_-FA, UiO-66-NO_2_-Teno, and UiO-66-NO_2_. Protein solutions were again prepared in water (200 μg/mL), and the compounds were tested with 60 μg of the protein. The Annexin-g03104 and SARS S-protein used in these experiments are non-glycosylated as a consequence of recombinant heterologous expression in *Escherichia coli*.

We found significant protein binding (65%) to both Annexin-g03104 and SARS S-protein for the three best-performing MOFs, despite the proteins having very different protein sequences and as such secondary and tertiary structures (Figure 2b). The extent of binding by the MOFs is different for Annexin-g03104 and SARS S-protein. For Annexin-g03104, binding is highest for UiO-66-NO_2_-FA (99%) and equivalent amounts for UiO-66-NO_2_ (69.5%) and UiO-66-NO_2_-Teno (65%), whereas for SARS S-protein binding, the ranking of the three MOFs is UiO-66-NO_2_ (100%) > UiO-66-NO_2_-FA (85%) > UiO-66-NO_2_-Teno (71%). In terms of comparison between antiviral agents, although both FA- and Teno-functionalized MOFs have a similar extent of binding for BSA, the FA-modified MOF has a relatively higher affinity for both Annexin and SARS S-protein over Teno-functionalized UiO-66-NO_2_. The differences in protein binding are thus not only ascribed to variation of functional groups but also ascribed to the distinct surface charges of the MOF nanoparticles (Figure S18). Surface charges have been seen to affect the binding of proteins in different materials^49,50^ and may also be a contributing factor for the functionalized UiO-66-NO_2_ MOFs to display higher binding affinity.

## 4 Conclusions

In summary, we successfully introduced highly porous MOFs as promising materials for combating the transmission of airborne viruses, such as coronaviruses. The postsynthetic modification of three Zr(IV)-based UiO-66 MOFs, namely, UiO-66, UiO-66-NH_2_, and UiO-66-NO_2_, with three antiviral agents, i.e., nystatin, folic acid, and tenofovir, turned out to be extremely powerful to boost the binding affinity toward several proteins, such as glycosylated BSA, non-glycosylated Annexin-g03104, and the spike protein of SARS. Moreover, the internal pores of the MOFs are accessible to water even after functionalization, which is expected to cause local dehydration and inactivate viruses. Our proof-of-concept study paves the way for further investigations with different functional drug molecules and MOF structures, in addition to combining these with fabrics. Based on the current findings, we believe that the approach will not only open a new frontier in the domain of MOFs and porous materials but also spur interest beyond the scope of PPEs such as in engineering problems like indoor air purification or biomedical applications.

## Supporting Information Available

PXRD patterns; FTIR spectra; UV–vis spectra; Raman spectra; SEM images; TGA profiles; gas adsorption isotherms; water adsorption isotherms; and ζ potential measurement (PDF)

## Notes

The research data underpinning this work can be accessed at doi.org/10.17630/f2143101-255b-43f4-ab4e-1e0037319f9e.
