# Supplementary material for: Surface-Functionalized Metal–Organic Frameworks for Binding Coronavirus Proteins
Source: ACS Appl Mater Interfaces. 2023 Feb 14;15(7):9058–65. doi: 10.1021/acsami.2c21187 (PMC9940617; doi:10.1021/acsami.2c21187)
Supplement: Supplementary file 1 — am2c21187_si_001.pdf [file am2c21187_si_001.pdf]

Supporting Information

Surface functionalized metal-organic frameworks for binding coronavirus proteins

Aamod V. Desai,,[I] Simon M. Vornholt,[I] Louise L. Major,[‡] Romy Ettlinger,[I] Christian Jansen,[#] Daniel N. Rainer,[I] Richard de Rome,[I] Venus So,[I] Paul S. Wheatley,[I] Ailsa K. Edward,[I] Caroline G. Elliott,[I] Atin Pramanik,[I] Avishek Karmakar,[^] A. Robert Armstrong,[I] Christoph Janiak,[#] Terry K. Smith[I,‡] and Russell E. Morris*,[I]*

[I] *- EastChem School of Chemistry, University of St Andrews, North Haugh, St Andrews KY16 9ST, United Kingdom*

[‡] *- School of Biology, University of St Andrews, Biomedical Sciences Research Complex North Haugh, St Andrews, KY16 9ST, United Kingdom*

[#] *- Institut für Anorganische Chemie und Strukturchemie, Heinrich-Heine-Universität Düsseldorf, 40204 Düsseldorf, Germany*

[^] *- Department of Chemistry, University of Pennsylvania, Philadelphia, PA-19104-6323, United States of America*

Email: avd6@st-andrews.ac.uk ; rem1@st-andrews.ac.uk

Figures

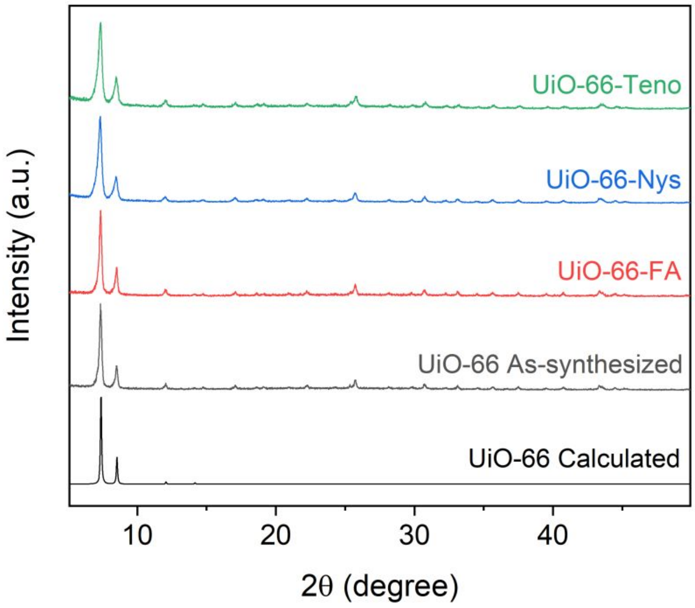

Figure S1. PXRD patterns for as-synthesized UiO-66 and post-synthetically modified products, compared to the calculated pattern for UiO-66 (Refcode: RUBTAK, reference [S1]).

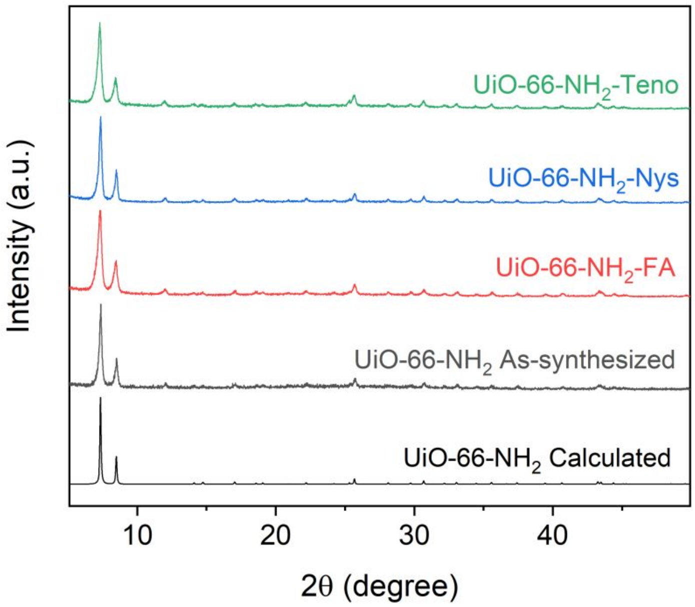

Figure S2. PXRD patterns for as-synthesized UiO-66-NH_2 and post-synthetically modified products, compared to the calculated pattern for UiO-66-NH_2 (Refcode: SURKAT, reference [S2]).

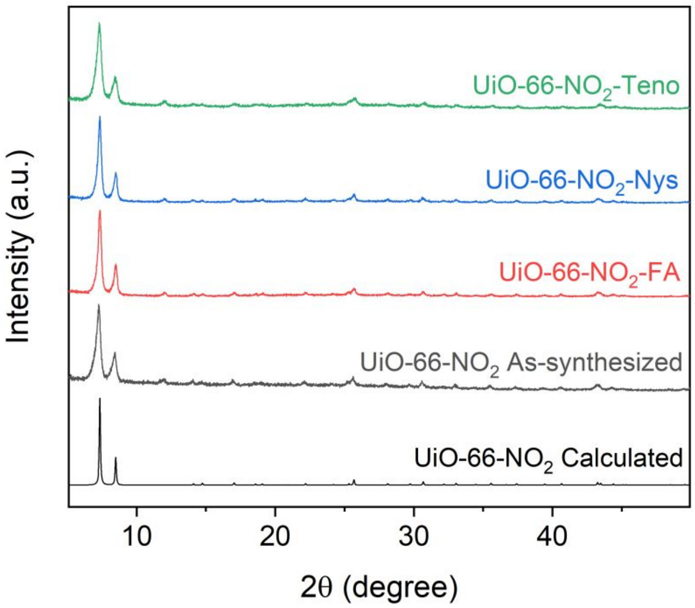

Figure S3. PXRD patterns for as-synthesized UiO-66-NO_2 and post-synthetically modified products, compared to the calculated pattern for UiO-66-NO_2 (Refcode: SURKEX, reference [S2]).

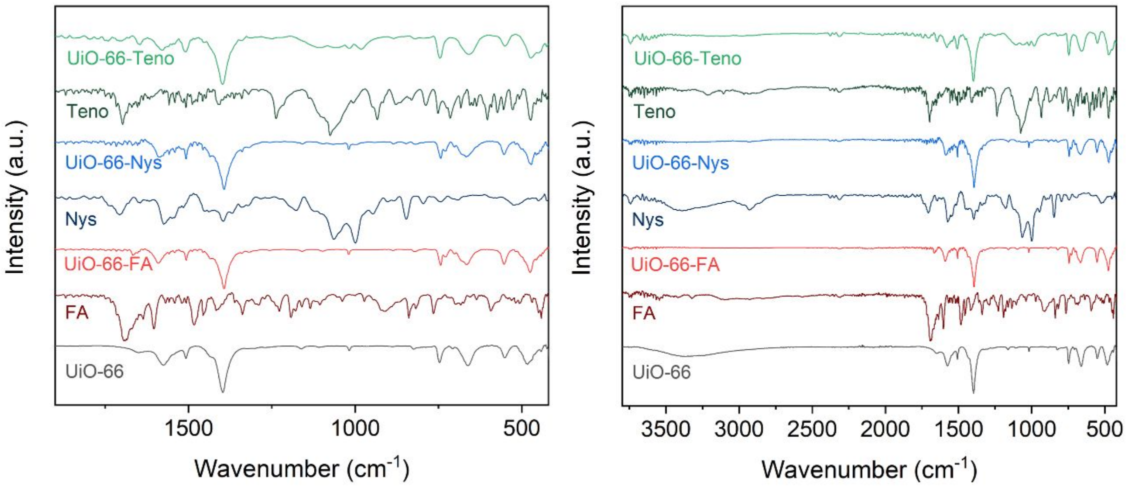

Figure S4. (Left) Zoomed and (right) full FTIR spectra for UiO-66 and functionalized products compared to individual tag molecules – FA (folic acid), Nys (nystatin) and Teno (tenofovir).

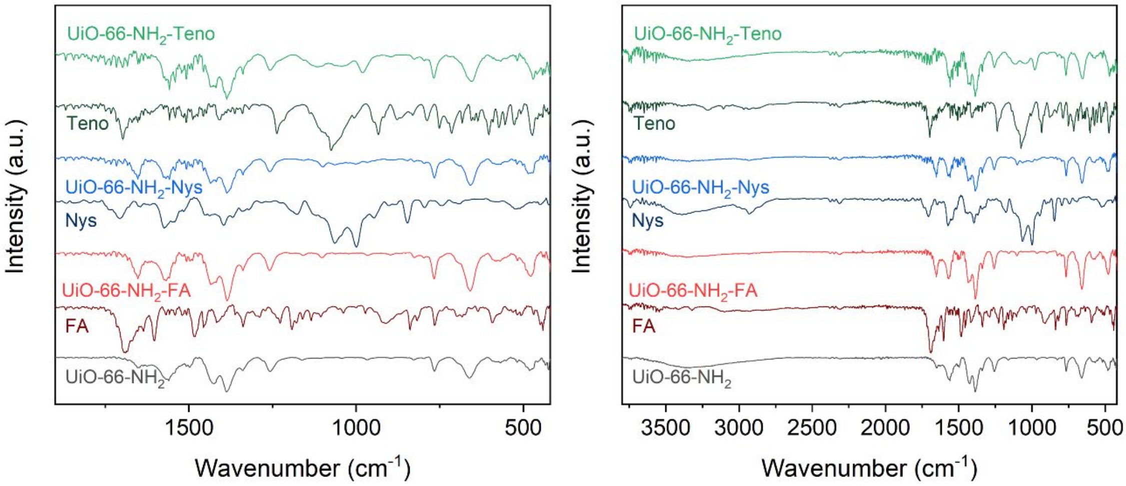

Figure S5. (Left) Zoomed and (right) full FTIR spectra for UiO-66-NH$_2$ and functionalized products compared to individual tag molecules – FA (folic acid), Nys (nystatin) and Teno (tenofovir).

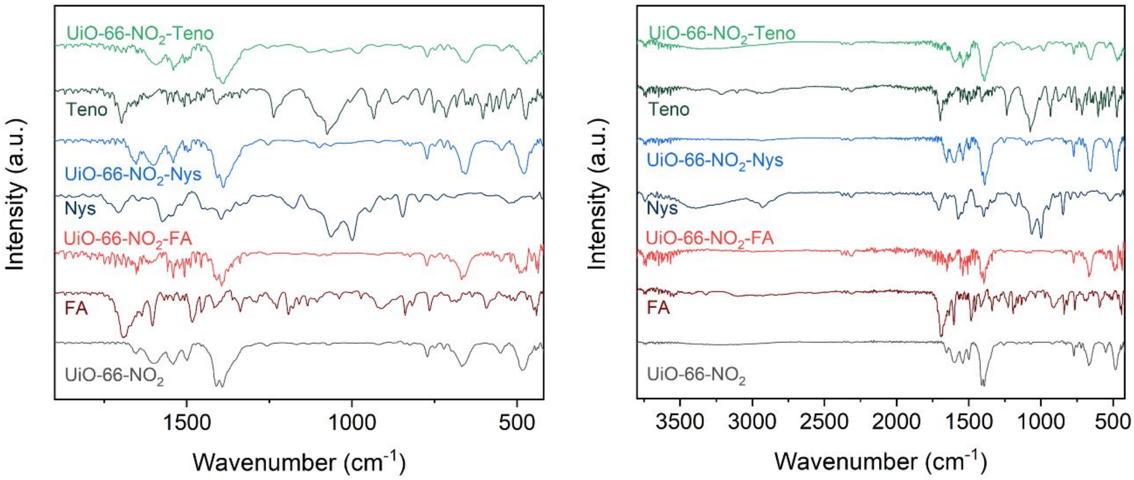

Figure S6. (Left) Zoomed and (right) full FTIR spectra for UiO-66-NO$_2$ and functionalized products compared to individual tag molecules – FA (folic acid), Nys (nystatin) and Teno (tenofovir).

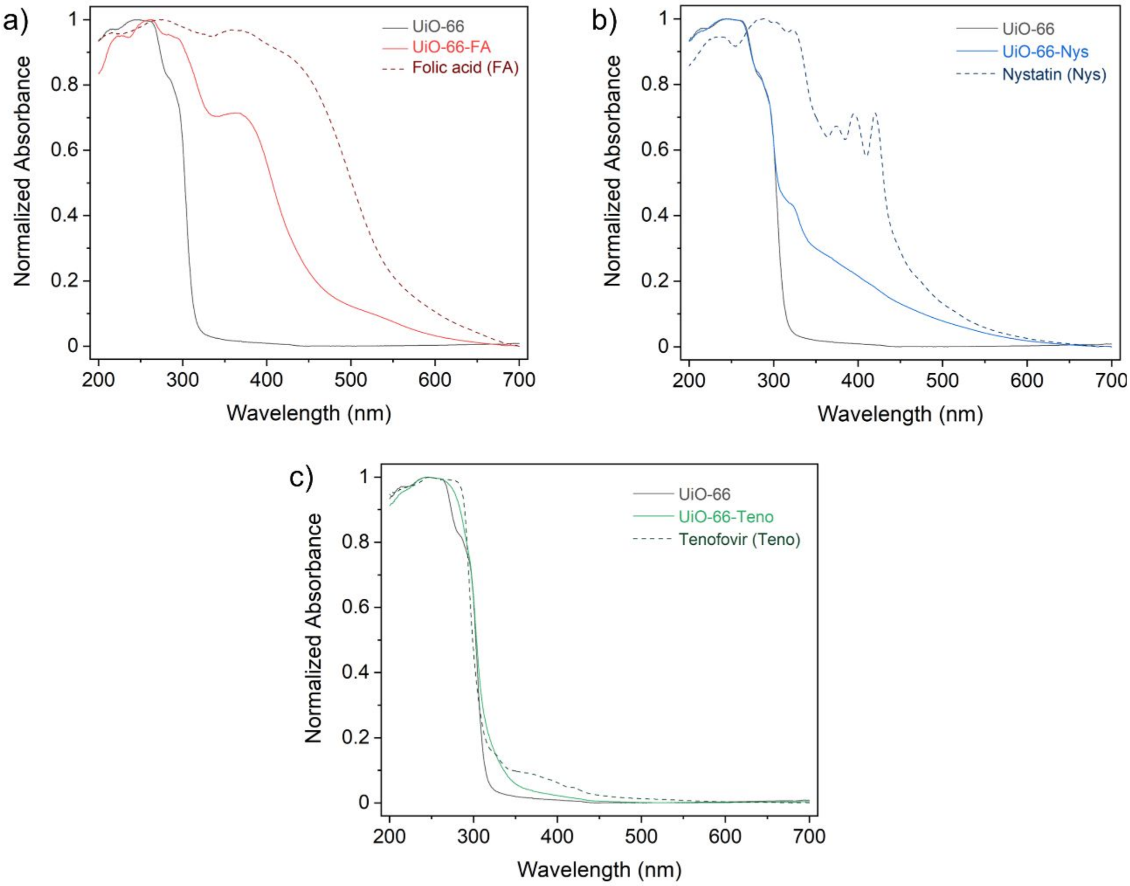

Figure S7. Normalized solid-state UV/vis diffuse-reflectance spectra for UiO-66 and functionalized products – a) folic acid and UiO-66-FA, b) nystatin and UiO-66-Nys, c) tenofovir and UiO-66-Teno.

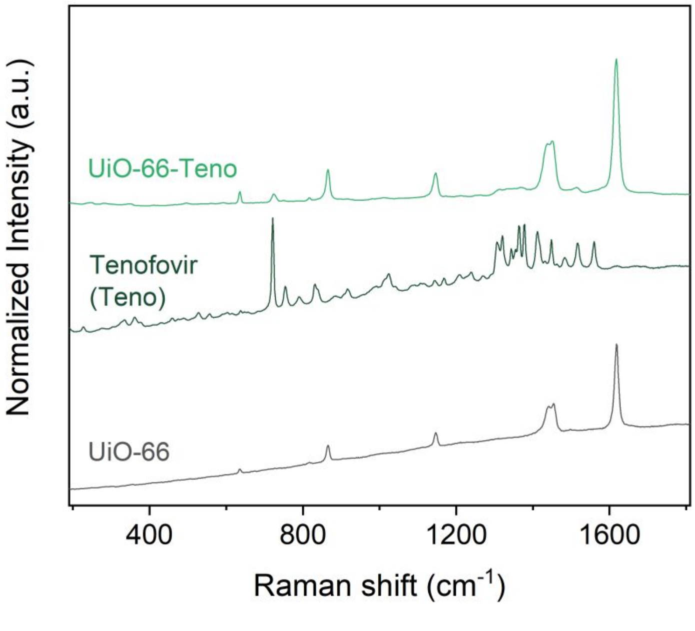

Figure S8. Raman spectra for UiO-66, tenofovir (Teno) and UiO-66-Teno (laser source – 532 nm).

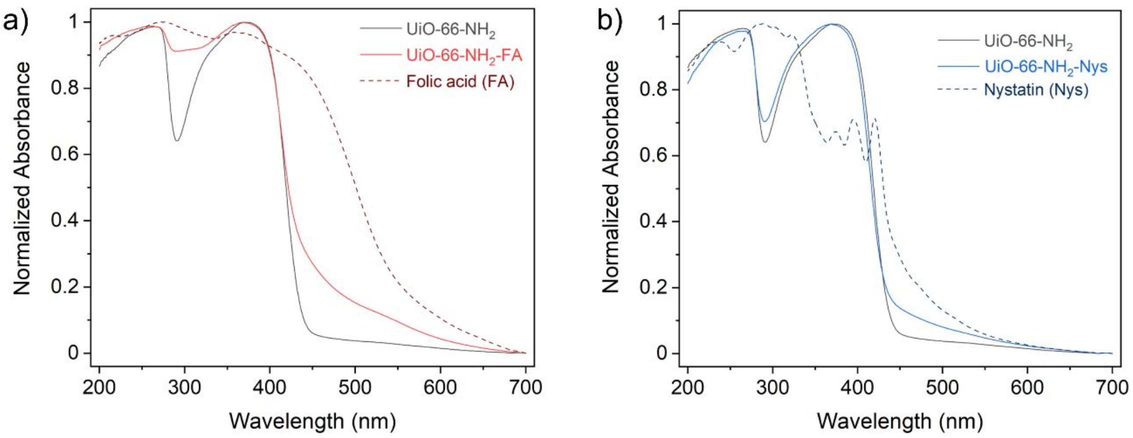

Figure S9. Normalized solid-state UV/vis diffuse-reflectance spectra for UiO-66-NH$_2$ and functionalized products – a) folic acid and UiO-66-NH$_2$-FA, b) nystatin and UiO-66-NH$_2$-Nys.

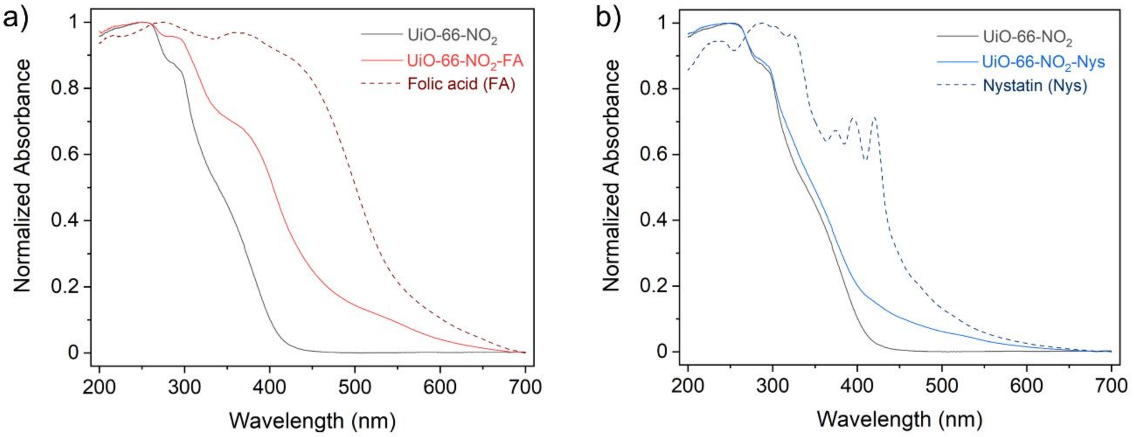

Figure S10. Normalized solid-state UV/vis diffuse-reflectance spectra for UiO-66-NO$_2$ and functionalized products – a) folic acid and UiO-66-NO$_2$-FA, b) nystatin and UiO-66-NO$_2$-Nys.

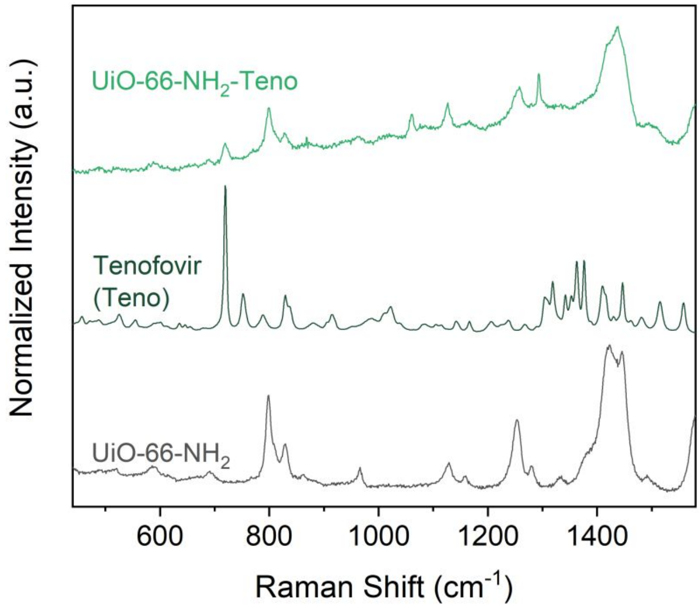

Figure S11. Raman spectra for UiO-66-NH$_2$, tenofovir (Teno) and UiO-66-NH$_2$-Teno (laser source – 785 nm).

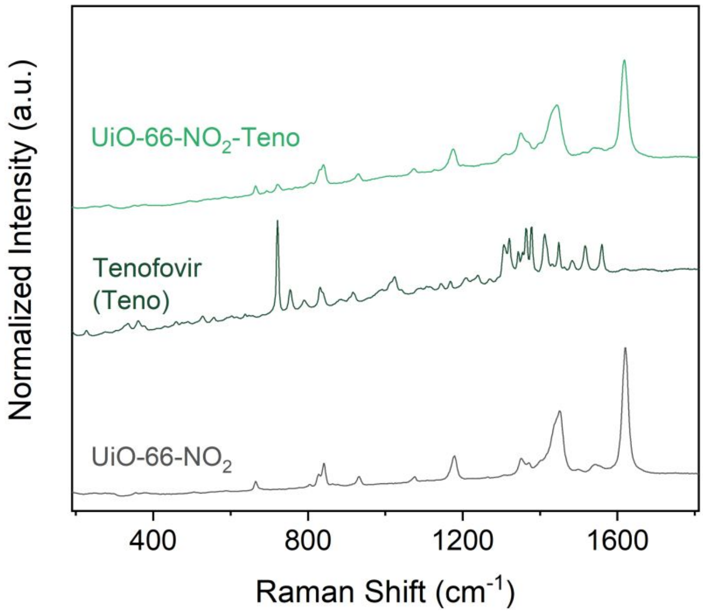

Figure S12. Raman spectra for UiO-66-NO$_2$, tenofovir (Teno) and UiO-66-NO$_2$-Teno (laser source – 532 nm).

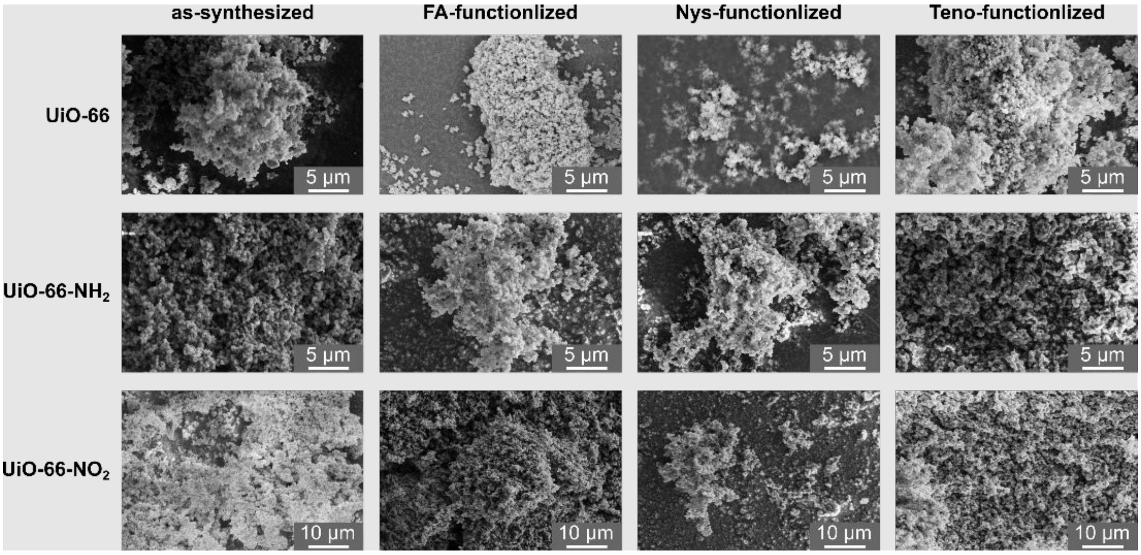

Figure S13. SEM images for all the compounds studied in this work – pristine MOFs (UiO-66, UiO-66-NH_2 and UiO-66-NO_2) and functionalized compounds with FA, Nys and Teno.

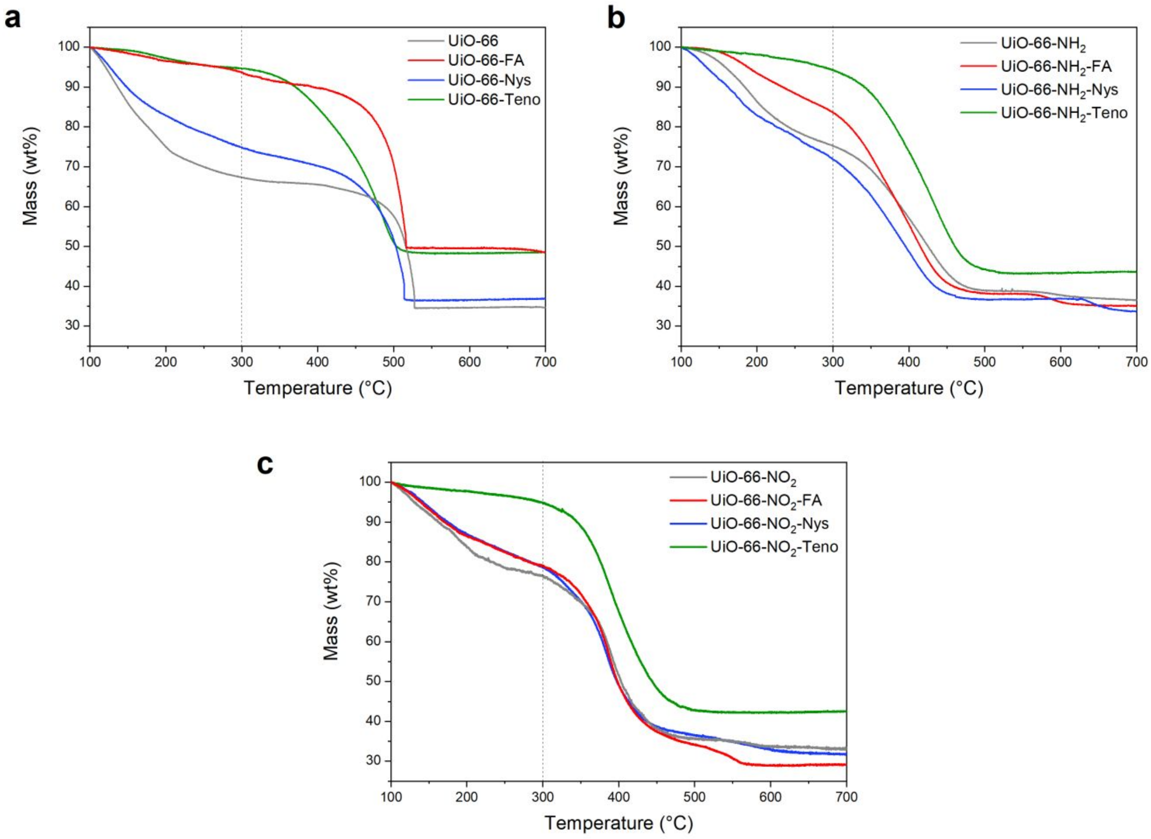

Figure S14. TGA profiles between 100 and 700°C for pristine MOFs and respective functionalized products - a) UiO-66, b) UiO-66-NH_2, c) UiO-66-NO_2. For a better comparison of their decomposition profiles, the release of different amounts of water due to their respective water adsorption behaviours below 100 °C was omitted.

Table S1. Analysis of the weight loss (wt%) between 300 and 700°C of UiO-66, UiO-66-NH_2, UiO-66-NO_2, and their functionalized analogues, including the determined amount of each functionalization, i.e., $\Delta_{Funct.-Pristine}$.

**Functionalization**	**Weight Loss 300-700 °C (wt%)**					
	**UiO-66**	**$\Delta_{Funct.-Pristine}$**	**UiO-66-NH_2**	**$\Delta_{Funct.-Pristine}$**	**UiO-66-NO_2**	**$\Delta_{Funct.-Pristine}$**
Pristine	32.5	–	38.5	–	43.9	–
FA	44.9	12.4	48.5	10.0	46.6	2.7
Nys	38.3	5.8	38.5	± 0	49.9	6.0
Teno	46.0	13.5	50.5	12.0	52.2	8.3

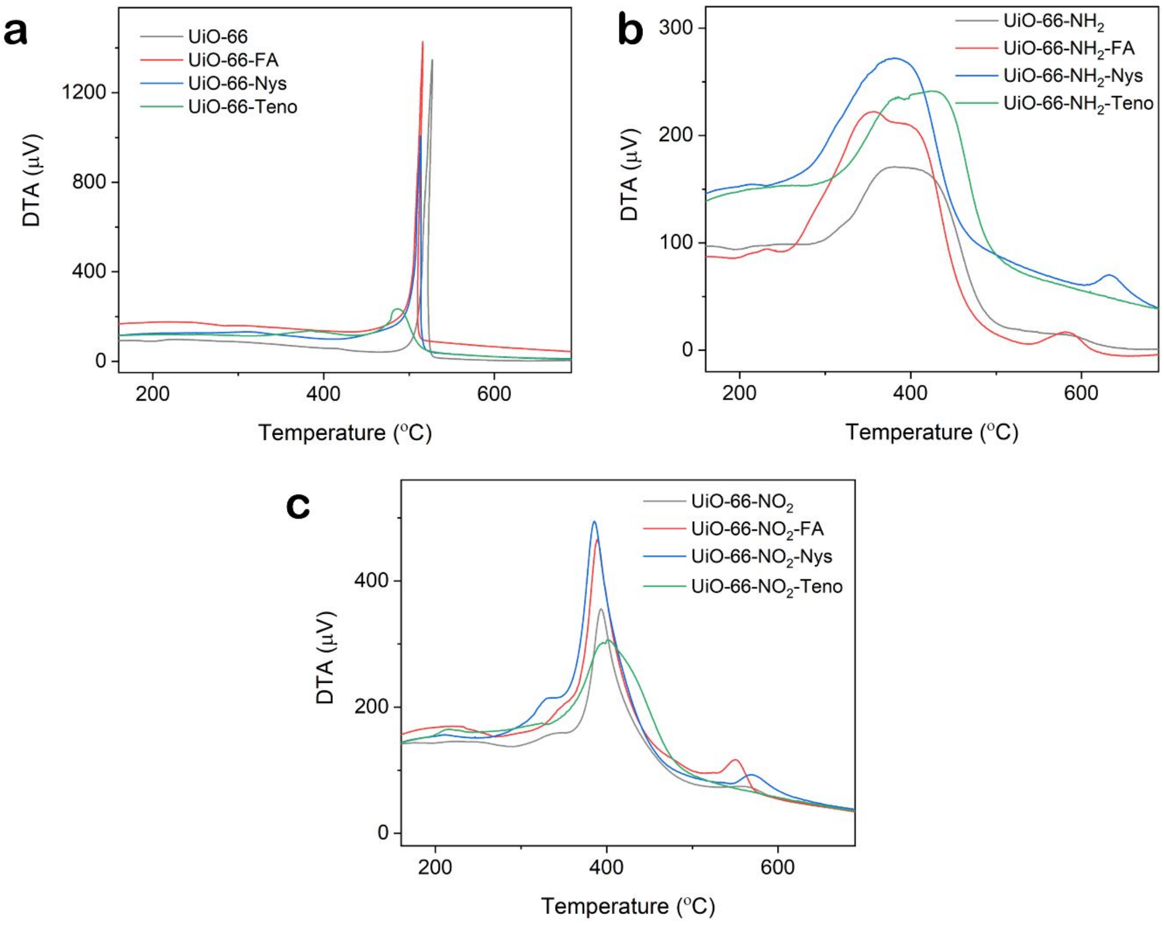

Figure S15. DTA profiles for pristine MOFs and respective functionalized products - a) UiO-66, b) UiO-66-NH_2, c) UiO-66-NO_2.

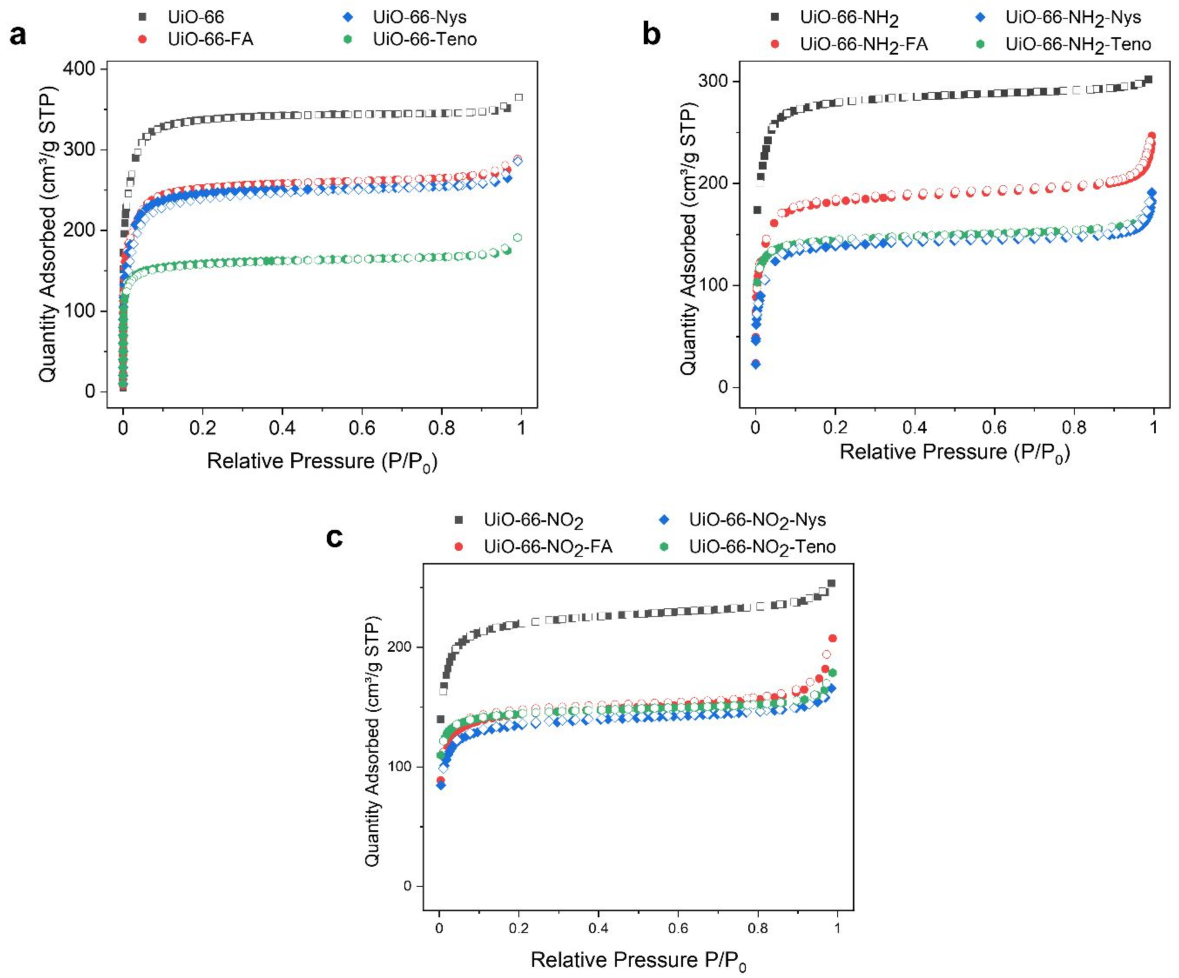

Figure S16. N_2 adsorption (77 K) isotherms for a) UiO-66, b) UiO-66-NH_2, and c) UiO-66-NO_2 with pristine MOFs (grey) and respective functionalized products -FA (red), -Nys (blue) and -Teno (green). Open and closed symbols represent adsorption and desorption respectively.

Table S2. Brunauer-Emmett-Teller (BET) surface areas for UiO-66, UiO-66-NH_2, and UiO-66-NO_2 and their functionalized compounds obtained using N_2 adsorption isotherms at 77 K.[a]

Compound	wt% UiO-66-X [b]	BET surface area (m^2/g) (estimate, composite)	Pore volume (cm^3/g) [d]
UiO-66	100	1288	0.53
UiO-66-FA	87.6	952 (1128)	0.39
UiO-66-Nys	94.2	950 (1213)	0.38
UiO-66-Teno	86.5	620 (1114)	0.24
UiO-66-NH_2	100	1151	0.43
UiO-66-NH_2-FA	90.0	733 (1036)	0.26
UiO-66-NH_2-Nys	100	562 (1151)	0.21
UiO-66-NH_2-Teno	88.0	579 (1013)	0.22
UiO-66-NO_2	100	891	0.34
UiO-66-NO_2-FA	97.3	575 (867)	0.21
UiO-66-NO_2-Nys	94.0	542 (838)	0.20
UiO-66-NO_2-Teno	91.7	577 (817)	0.22

[a] The BET surface areas of the composites must be compared to the expected estimated BET surface area of UiO-66-X which is corrected by the lower mass percent of UiO-66-X in the composite according to:

$$\text{BET (estimated,composite)} = \frac{\text{wt\% of UiO} - 66}{100} \times 1288\, m^2 \cdot g^{-1}$$

and alike for UiO-66-NH_2 and UiO-66-NO_2.

[b] From the TGA data in Table S1 we take the added weight loss in wt% as the grafted amount on the MOF surface and its difference to 100% as the wt% of UiO-66-X

[c] From the formula in Table footnote a and the wt% UiO-66-X in the second column the theoretically achievable surface area for the lower UiO amount in the composite is obtained.

[d] Pore volume at $P/P_0 = 0.95$.

Comment to Table S2: The comparison between the experimental BET surface area and the estimate for the composite based upon the wt% of UiO-66-X reveals considerable pore blocking effect. The strongest pore blocking effects are seen for UiO-66-Teno, UiO-66-NH_2-Nys (despite its very small loading) and UiO-66-NH_2-Teno.

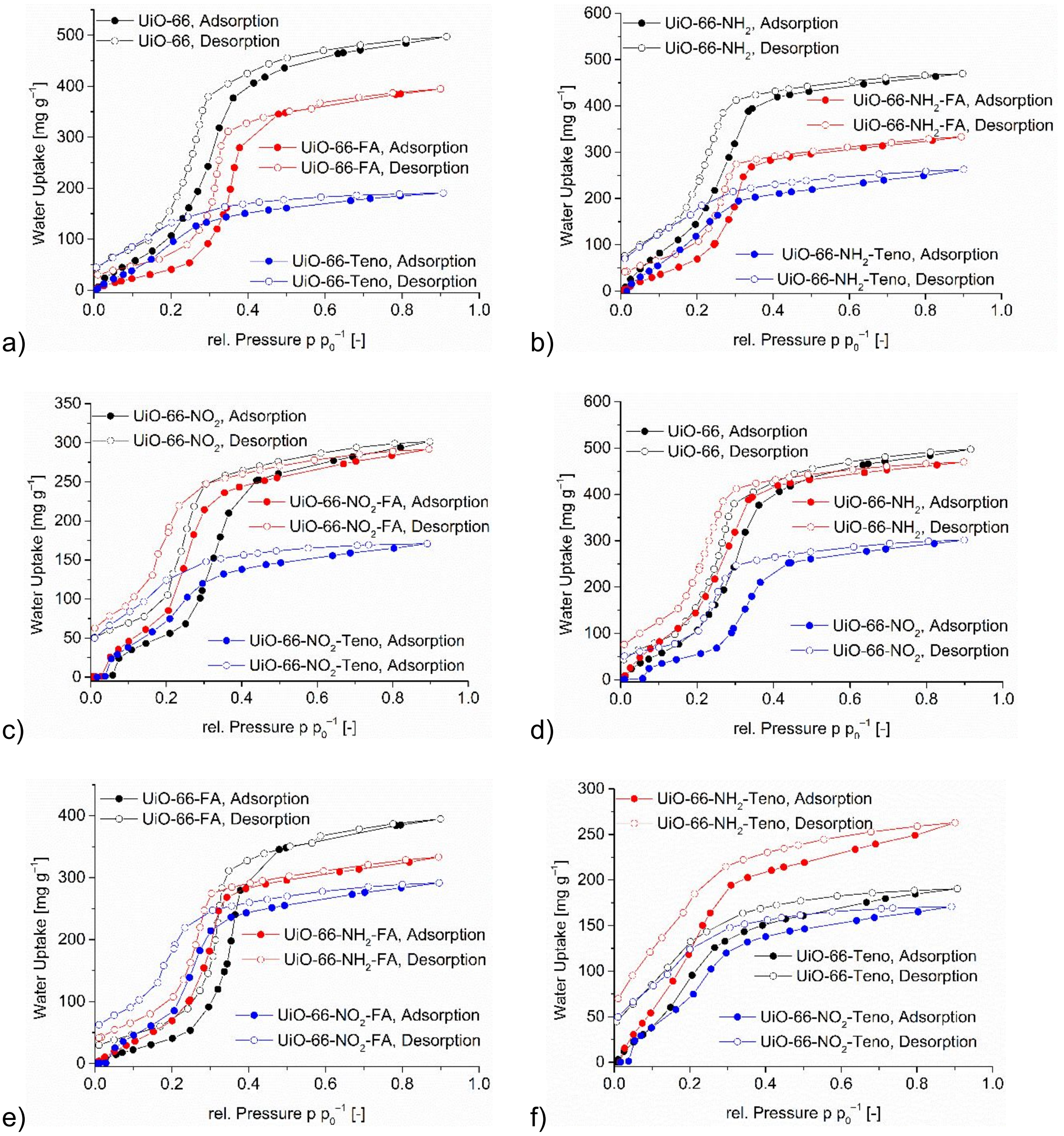

Figure S17. (a-c) Water adsorption isotherms at 298 K for parent MOFs and composites with FA and Teno – (a) UiO-66; (b) UiO-66-NH_2 and (c) UiO-66-NO_2. (d) Water sorption isotherms for UiO-66, UiO-66-NH_2 and UiO-66-NO_2. (e-f) Water adsorption isotherms at 298 K for the UiO-66-X MOFs with the same drug molecules (e) folic acid and (f) tenofovir (Teno) (X = –, NH_2, NO_2).

Table S3: MOFs and MOF-composites with their specific uptake at different relative pressures.

MOF/MOF-Composite	Water uptake [mg g^{-1}] at given p p_0^{-1}				Water uptake estimate [mg g^{-1}] at 0.9 [a]
	**0.1**	**0.2**	**0.4**	**0.9**	
UiO-66	55	106	398	495	
UiO-66-FA	23	40	293	395	433
UiO-66-Teno	39	92	151	190	428
UiO-66-NH_2	81	150	415	470	
UiO-66-NH_2-FA	35	69	283	334	423
UiO-66-NH_2-Teno	56	121	209	263	414
UiO-66-NO_2	32	54	229	302	
UiO-66-NO_2-FA	46	83	244	292	294
UiO-66-NO_2-Teno	38	71	138	171	277

[a] The water uptake of the composites at the given relative pressure must be compared to the expected estimated water uptake of UiO-66-X which is corrected by the lower mass percent of UiO-66-X in the composite according to:

$$\text{water uptake (estimated,composite)} = \frac{\text{wt\% of UiO} - 66 - \text{X}}{100} \times \text{(water uptake) mg} \cdot g^{-1}$$

From the TGA data in Table S1 we take the added weight loss in wt% as the grafted amount on the MOF surface and its difference to 100% as the wt% of UiO-66-X (cf. column 2 in Table S2) Thereby the theoretically achievable water uptake for the lower UiO amount in the composite is obtained.

Comment to Table S3: The comparison between the experimental water uptake at p p_0^{-1} = 0.9 and the estimate for the composite, based upon the wt% of UiO-66-X, reveals considerable pore blocking effects for the Teno-functionalized compounds UiO-66-Teno, UiO-66-NH_2-Teno and UiO-66-NO_2-Teno.

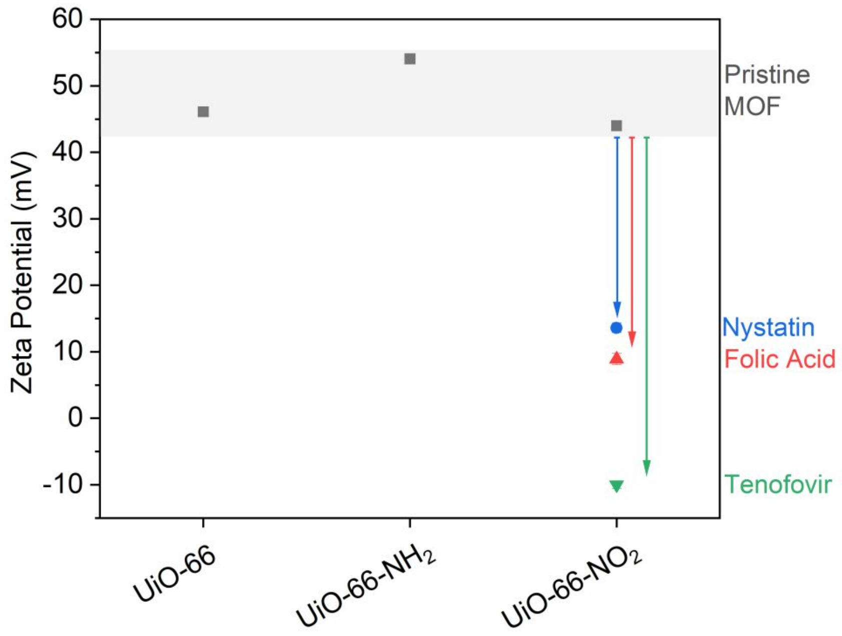

Figure S18. Zeta potential for parent MOFs and UiO-66-NO_2 composites with Nys, FA and Teno.

References:

(1) Cavka, J. H.; Jakobsen, S.; Olsbye, U.; Guillou, N.; Lamberti, C.; Bordiga, S.; Lillerud, K. P. A New Zirconium Inorganic Building Brick Forming Metal Organic Frameworks with Exceptional Stability. *J. Am. Chem. Soc.* **2008**, *130* (42), 13850–13851. https://doi.org/10.1021/ja8057953.

(2) Trickett, C. A.; Gagnon, K. J.; Lee, S.; Gándara, F.; Bürgi, H.-B.; Yaghi, O. M. Definitive Molecular Level Characterization of Defects in UiO-66 Crystals. *Angew. Chemie Int. Ed.* **2015**, *54* (38), 11162–11167. https://doi.org/https://doi.org/10.1002/anie.201505461.
